# Editorial Comment to Possible abscopal effect in urothelial carcinoma of the upper urinary tract after treatment with immune checkpoint inhibitors

**DOI:** 10.1002/iju5.12136

**Published:** 2019-12-12

**Authors:** Takeshi Yuasa

**Affiliations:** ^1^ Department of Urology Cancer Institute Hospital Japanese Foundation for Cancer Research Tokyo Japan

The abscopal effect was first described in 1953 by Dr Mole as “an effect of ionizing radiation at a distance from the irradiated volume but within the same organism,” and it now refers to shrinkage of non‐irradiated primary and/or metastatic lesions following radiotherapy.^1^ Although the precise mechanism of the abscopal effects remains unclear, an increasing number of recent reports in the immune checkpoint inhibitors era support that activation of the immune system and modulation of the tumor microenvironment by radiation and immune checkpoint inhibitor therapy likely plays important roles in this treatment effect.^2^

In this issue of *IJU Case Reports*, Ishiyama *et al*. reported the case of a patient with renal pelvic cancer who had recurrence after radical nephroureterectomy.^3^ After gemcitabine and cisplatin combination chemotherapy followed by pembrolizumab immune therapy, the patient underwent radiation therapy to treat the local recurrence lesion and for pain relief.^3^ With dramatic symptom relief, computed tomography demonstrated a reduced size of the irradiated and multiple non‐irradiated lymph nodes.^3^ The authors considered this case to be an abscopal effect after treatment with an immune checkpoint inhibitor and they concluded that this combined and/or sequential administration of an immune checkpoint inhibitor and radiation therapy may become a new metastatic urothelial cancer treatment strategy.^3^

Based on the promising anti‐tumor efficacy and a manageable safety profile, pembrolizumab therapy has been rapidly introduced and the paradigm of medical treatment for metastatic urothelial cancer is dramatically changing in clinical practice in Japan.^4^ In addition to the agent's intrinsic efficacy, the abscopal effect that is induced by radiation therapy is recognized in various malignancies in this immune checkpoint inhibitor era, although the incidence and radiation characteristics including dose, fraction, and timing required for its occurrence remain unknown.^2^ In a single institutional retrospective study, Trommer *et al*. investigated the characteristics of the abscopal effect among the 126 patients who received an immune checkpoint inhibitor and radiation therapy. Twenty‐four of these patients had eligible non‐irradiated lesions and an abscopal effect was observed in seven patients (29%, malignant melanoma: *n* = 3, non‐small cell lung cancer: *n* = 3, renal cell carcinoma: *n* = 1).^5^ This suggests that the abscopal effect might not be a rare event in the immune checkpoint inhibitor era.^5^ When the precise mechanism and the optimal radiation characteristics are revealed, the abscopal effect may be incorporated into the treatment strategy as an important option in metastatic urothelial cancer.

## Conflict of interest

The author declares no conflict of interest.

## References

[1] Mole RH . Whole body irradiation; radiobiology or medicine? Br. J. Radiol. 1953; 26: 234–41. 13042090 10.1259/0007-1285-26-305-234

[2] Reynders K , Illidge T , Siva S , Chang JY , De RD . The abscopal effect of local radiotherapy: using immunotherapy to make a rare event clinically relevant. Cancer Treat. Rev. 2015; 41: 503–10. 25872878 10.1016/j.ctrv.2015.03.011 PMC4816218

[3] Ishiyama Y , Takagi T , Yoshida K *et al* Possible abscopal effect in urothelial carcinoma of the upper urinary tract after treatment with immune checkpoint inhibitors. IJU Case Rep. 2020; 3: 25–7. 10.1002/iju5.12133 PMC7292173 32743462

[4] Yuasa T , Urakami S , Yonese J . Recent advances in medical therapy for metastatic urothelial cancer. Int. J. Clin. Oncol. 2018; 23: 599–607. 29556919 10.1007/s10147-018-1260-0 PMC6097083

[5] Trommer M , Yeo SY , Persigehl T *et al* Abscopal effects in radio‐immunotherapy response analysis of metastatic cancer patients with progressive disease under anti‐PD‐1 immune checkpoint inhibition. Front Pharmacol. 2019; 10: 511. 31156434 10.3389/fphar.2019.00511 PMC6530339

